# The impact of vaccine information and other factors on COVID-19 vaccine acceptance in the Thai population

**DOI:** 10.1371/journal.pone.0276238

**Published:** 2023-03-07

**Authors:** Hathairat Kosiyaporn, Chanikarn Netrpukdee, Nattanicha Pangkariya, Orana Chandrasiri, Viroj Tangcharoensathien

**Affiliations:** International Health Policy Program, Ministry of Public Health, Nonthaburi, Thailand; Johns Hopkins University School of Medicine, UNITED STATES

## Abstract

Increased misinformation circulating among the population during the COVID-10 pandemic can trigger rejection to take up vaccines. This study assesses the influence of vaccine information and other factors on vaccine acceptance in the Thai population. Between March and August 2021, six rounds of cross-sectional surveys through village health volunteer networks and online channels were conducted; as well as qualitative interviews with frontline health workers, patients with chronic diseases, and religious believers and leaders. Descriptive and multiple logistic regression with 95% level of confidence were used for survey findings while deductive thematic analysis was used for in-depth interview findings. Among the total 193,744 respondents, the initial COVID-19 vaccine acceptance rate decreased from 60.3% in March 2021 to 44.0% in April 2021, then increased to 88.8% in August 2021. Participants who were able to differentiate true and false statements were 1.2 to 2.4 times more likely to accept vaccine than those who were not. Those who perceived a high risk of infection (Adjusted odds ratio; AOR = 2.6–4.7), perceived vaccine safety (AOR = 1.4–2.4), judged the importance of vaccination (AOR = 2.3–5.1), and had trust in vaccine manufacture (AOR = 1.9–3.2) were also more likely to accept the vaccine. Moreover, higher education (AOR = 1.6–4.1) and living in outbreak areas (AOR = 1.4–3.0) were significantly related to vaccine uptake, except in people with chronic diseases who tended not to accept the vaccine (AOR = 0.7–0.9). This study recommends effective infodemic management and comprehensive public communication, prioritising vulnerable groups such as those with a low level of education and people with chronic conditions. Communication through reliable channels can support higher vaccine acceptance and rapid vaccine rollout. Finally, regular monitoring of misinformation is important such as fact checking support, timely legal actions and specific debunking communication.

## Introduction

Misinformation on COVID-19 vaccines can trigger vaccine hesitancy and hampers government efforts to increase coverage in order to minimize mortality. Those who are exposed to misinformation about vaccines tend to believe in fake news and conspiracy theories and go on to reject the vaccine [1,2]. Vaccine information or ‘Communication in five Cs’ concept is applied to explain vaccine acceptance in the population using Confidence, Complacency, Convenience, Context and Communication [3]. The nature of the COVID-19 vaccine, such as its novelty, uncertainties of efficacy, complications and adverse events leads to higher vaccine refusal in the population [4]. The World Health Organization (WHO) defines an overwhelming quantity of accurate or inaccurate information as ’infodemic’ [5] which is increasingly significant especially in the era of digital disruption.

Previous studies about vaccine hesitancy were conducted in both exploratory and explanatory studies. Most were cross-sectional surveys in the general population or certain population such as health professionals; and mostly took place in high-income countries [6,7]. There are limited studies on vaccine acceptance in low- and middle- income countries. Personal characteristics such as gender, age, education, and occupation are associated with vaccine acceptance. Further, trust in government institutions, perceived risk of COVID-19 infection, vaccine efficacy and safety [6,7] also play key roles. Studies show that low socioeconomic status and self-identifying as belonging to a minority group is significantly associated with higher susceptibility to misinformation or belief in fake news leading to vaccine refusal [1,8].

Thailand’s first report of a confirmed COVID-19 case outside China triggered public health and social measures to contain the pandemic led by a comprehensive multi-sectoral response [9,10]. Since February 2020, there have been four waves of the outbreak. The third and fourth waves in 2021 were the most serious due to the Delta strain with high mortality, followed by Omicron at the end of 2021 and continuing to 2022 [11]. Vaccine rollout, targeting healthcare workers, was launched in February 2021 and was extended to high-risk groups such as the elderly, obese, and patients with chronic diseases in June 2021. At the beginning, the government principally provided two vaccines: Sinovac and AstraZeneca, as approved by the Thai Food and Drug Administration [12]. Information (and misinformation) about the vaccine rapidly emerged alongside the vaccine rollout.

A few cross-sectional online studies in Thailand identified the reasons for vaccine refusal [13–16]. The vaccine acceptance rate increased in May 2021, with the population most concerned about the risks and benefits of vaccination [15]. The emergence of the COVID-19 Delta strain in August 2021, with higher case fatality, and the provision of vaccine information on safety, efficacy and availability influenced people’s decision to vaccinate [17]. A study by WHO on ‘Early artificial intelligence-supported response with social listening’ monitored social trends related to COVID-19 in Thailand from Twitter, Facebook, news comments and blogs. It showed that vaccination was the most popular topic at that time [18]. Another social listening study by a social media analytic company showed that at the beginning of vaccine rollout, Thai people mostly discussed the side effects of vaccines followed by vaccine communication and management [19]. Therefore, accurate and transparent vaccine information plays a critical role for vaccine uptake in the population.

There have been no large-scale surveys of vaccine acceptance in the Thai population. Prior studies have not addressed misinformation and vaccine acceptance or included those who do not have access to the Internet. To inform the operation of the vaccine rollout and support effective infodemic management, this study assessed the vaccine acceptance rate, and the influence of vaccine information (both accurate and mis-information) and other factors on vaccine acceptance in the Thai population.

## Materials and methods

**Fig 1:**
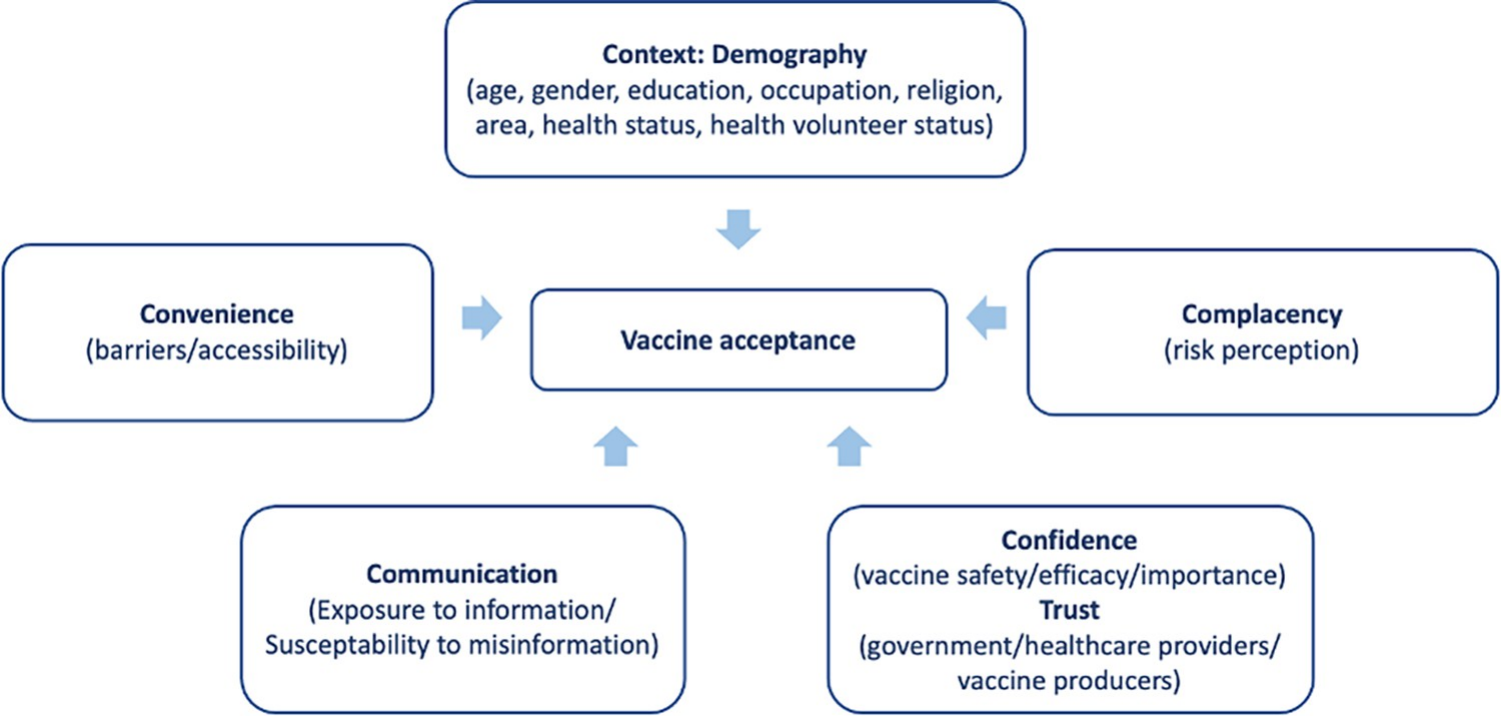
Conceptual framework.

We developed a conceptual framework modified from Razai et al. and Figueiredo et al. [3,20] where vaccine acceptance, refusal and hesitance are key dependent variables, and others such as demographic characteristics, perceived risks, confidence in vaccine safety, efficacy and the importance of vaccination, trust in government institutions, healthcare providers, vaccine manufacturers, and vaccine information are independent variables that can concertedly influence the population’s decision to receive vaccines. The convenience or barriers to access vaccines are excluded from the discussion in this paper. See Fig 1.

### Study design

A mixed-method design was employed. A series of cross-sectional surveys and key informant interviews with was conducted between March and August 2021. Survey respondents were the general Thai population, while key informants from interviews were specific samples who live in geographical areas classified by the level of risks of outbreak.

### Participants and data collection

For quantitative data, six rounds of surveys were conducted among the general public aged over 15 years old. The research team worked closely with the Department of Health Service Support, Ministry of Public Health (MOPH) to integrate this module with the ongoing survey "Stay Home, Save Lives", which aims to monitor people’s compliance on the disease control measures. Survey data were collected fortnightly through the network of village health volunteers (VHVs) in all 77 provinces using Google Form. Rounds 1, 2, 3, 4, 5 and 6 were conducted in 1–15 March, 16–30 April, 16–31 May, 16–30 June, 16–31 July, 16–31 August of 2021. Each round took 15 days before closing the survey for analysis.

In 2020, Thailand had more than 1.05 million VHVs nationwide. They are recruited by local communities and trained by MOPH, and each is responsible for 10–15 households in their community [21]. Since 2019, the monthly honorarium has been increased to 1000 Baht (US$ 33) to each VHV supported by the government and additional 500 Baht (US$ 16) for six months to support their contribution to COVID-19 containment during the pandemic [22].

The participants were conveniently selected based on their willingness to respond to the Google form disseminated by VHVs. We did not require VHVs to keep track of the same respondents to respond to the following survey rounds. Additionally, an online survey via Facebook or website was distributed to ensure additional coverage. Sample size was calculated from Yamane’s formula (n = N/1+Ne^2^). N stood for the range of population, 194,573 to 5,527,994 per province and e referred to 0.05; for which 400 samples per province are required [23]. To cover all 77 provinces, the total 30,700 samples per round (400 samples x 77 provinces), which is equivalent to total 184,800 samples in all six rounds (30,700 samples x 6 rounds) are required.

For the qualitative study, purposive sampling was conducted through online announcements and recommendations by the Provincial Islamic Committees, with the following inclusion criteria:

- The priority groups of vaccine rollout according to MOPH definition: health professionals, individuals with seven priority diseases (chronic respiratory disease, cardiovascular disease, end stage of chronic kidney disease, neurovascular disease, cancer, diabetes, obesity (weight > 100 kilograms or BMI > 35 kg/m^2^ [24]
- People who have high-risk occupations such as employees working in public spaces or the tourism sector, as they are the priority for the next phase of vaccine rollout [24]
- Those who were more likely to have low vaccine acceptance, identified from previous literature [25], such as Muslims in deep southern provinces.

These three groups of participants were recruited from low and high outbreak provinces [12]. The research team offered 500-Baht (US$ 16) compensation for their participation in the study.

### Measurement

The questionnaire comprised five sections: 1) demography (gender, age, education, occupation, place of residence, religion, health status, village health volunteer status (responses are multiple choices except age); 2) vaccine acceptance (multiple choices of yes/no/uncertainty); 3) have heard or seen information (multiple choices of yes/no) and ability to differentiate true and false statement (determining susceptibility of misinformation)(multiple choices of true/false/unsure); 4) trustworthy sources of information (Likert scale ranks from 1–5 from totally disagree, disagree, neutral, agree to totally agree); and 5) other factors related to vaccine acceptance such as self-perceived risk of infection, vaccine confidence, trust in the government, healthcare providers and vaccine manufacturers (Likert scale ranks from 1–5 from totally disagree, disagree, neutral, agree to totally agree) [8,20].

In each round of survey, there are five different true and false statements relating to the vaccine to assess participants’ ability to differentiate true and false information. These statements were generated from social listening by researchers and capture the dynamic of information flows on social media related to vaccines during vaccine rollout by the government. The process of selecting statements for each round of survey starts with monitoring headlines related to COVID-19 vaccine, then pick up the topics that were frequently mentioned or quoted by news agencies or widely discussed on social media platforms such as Facebook or Twitter. These findings were discussed among the research team and stakeholders, such as representatives from the local office of United Nations Children’s Fund (UNICEF) and WHO, and the Thai National Vaccine Institute and Department of Disease Control, MOPH to ensure validity and decide if the statement was true or false. It took 30–45 minutes to complete the self-administered questionnaire.

For qualitative data collection, the interview guide was adapted from the WHO Interim Guidance: for gathering and using data on the behavioural and social drivers of vaccination and the WHO European Region field guide to qualitative research for new vaccine introduction [26,27]. A semi-structured questionnaire comprising three main parts was developed, revised and finalised: 1) decision to vaccinate; 2) reasons to accept or refuse vaccine such as perceived risk of COVID-19 infection, vaccine confidence, trust in government and healthcare providers; and 3) what are the trustworthy sources of information? The interviews lasted approximately one hour via Zoom, Line, or by telephone, depending on the preferences of the interviewees. More than two researchers participated in each interview to avoid bias or domination; the conversation was electronically recorded after consent approval for detailed analysis.

### Data analysis

The quantitative surveys were analyzed by using STATA 14.0; descriptive and inferential statistics were applied. The characteristics of respondents, vaccine acceptance and trustworthy sources of information were presented in percentages while impact of the vaccine information and other related factors on vaccine uptake applied multivariable binary logistic regression with 95% confidence level.

In multivariable binary logistic regression analysis, only those who were exposed to information were analyzed because it is not valid to assess the capacity among participants who were not exposed to such information. The independent variable was demographic data whereas vaccine acceptance was a dependent variable. The other independent factors such as ability to differentiate true and false information, confidence in the vaccine, trust in the systems, and perceived risk of infection were analyzed separately with the same independent and dependent variables mentioned above.

Only high frequency of exposure and general knowledge statements, which were not limited to the Thai context, were selected and separately analyzed in each true and false statement (See S3 Table). Those who have the ability to differentiate information are defined as giving correct answers to the selected statements. The Likert scale of trustworthy sources of information, vaccine safety, effectiveness and importance, and trust in government, healthcare providers and vaccine producers were defined as high level if the answer is ‘agree’ or ‘strongly agree’ or score 4–5 [20].

In-depth interviews were transcribed and coded by the research team in an Excel program. A deductive analysis was employed using three main themes: 1) factors and their trade-off for making decision to vaccinate; 2) appraisal and trustworthy to sources of information; and 3) other factors related to vaccine uptake in specific groups such as religious belief.

### Ethical consideration

The research ethics of this study was approved by the Development of Human Research Protections (IHRP No. 059–2564). The participants and guardians/authorized representatives provided verbal informed consent before starting the surveys and interviews.

## Results

From the six rounds, there were altogether 193,744 respondents comprising 186,241 from the VHV network and 7,503 from an online survey. The number of participants decreased over the six rounds, the number of samples were 51,224 and 52,825 in the first two rounds, 26,795; 26,037 and 22,174 in the following three rounds and 14,689 in the last round.

More than three-quarters of participants were adults (75.8%) and female (79.5%); 44.5% of respondents had graduated from secondary school or had an equivalent qualification, while 33.5% had passed only primary education; 40.4% of participants worked in the agricultural sector, followed by 21.9% who identified as self-employed, daily workers and drivers. Most of them were from the north eastern region (35.3%), followed by the northern region (21.7%). When classifying four areas of COVID-19 cases, 34.4% of participants lived in minimum-risk provinces, 32.2% in moderate-risk provinces, 25.1% in high-risk provinces, and 8.3% in extreme high-risk provinces. A vast majority was Buddhist (94.0%) and around 5% were Muslim. 66.9% of the respondents were healthy while 26.7% had one of the seven priority chronic diseases for vaccination targets. Furthermore, around three-quarters of them were VHVs (72.3%). See Table 1. Thirty-six interviewees were recruited for in-depth interviews; the characteristics are described, see S1 Table.

**Table 1:** The characteristics of respondents combined in six rounds of surveys.

Demographic data	Subgroup	Number	Percentage
age (year)^β^	15–25	12,004	6.2
	26–59	146,941	75.8
	> 60	34,799	18.0
gender	male	38,443	19.8
	female	154,098	79.5
	Unspecified/missing	1,203	0.7
education level	lower or equal to primary school	64,995	33.5
	equal to secondary school	86,166	44.5
	equal to primary school	42,583	22.0
religion^β^	Buddhist	133,912	94.0
	Christian	1,219	0.9
	Muslim	6,766	4.7
	others/no religion	623	0.4
occupation	agricultural worker	78,288	40.4
	freelance/daily worker/driver	42,487	21.9
	unemployed/student	21,723	11.2
	employee in public and private sectors	22,483	11.6
	business owner	23,521	12.2
	health professional	5,242	2.7
region	central	32,638	16.9
	northern	42,056	21.7
	east-northern	68,451	35.3
	southern	25,202	13.0
	Bangkok and metropolitan	25,397	13.1
risk area^α^	Extreme high-risk provinces	15,985	8.3
	High-risk provinces	48,603	25.1
	Moderate-risk provinces	62,476	32.2
	Minimum-risk provinces	66,680	34.4
underlying disease^β^	healthy	95,361	66.9
	seven priority diseases	38,027	26.7
	other diseases	9,132	6.4
village health volunteer^β^	yes	103,050	72.3
	no	39,470	27.7
	**total**	193,744	100.0

^α^Based on the announcements of the government which ranged from extreme high-risk provinces (highest number of new cases, with full restrictions) to minimum-risk provinces (lowest number of new cases, with least restrictions) [12].

^β^Total respondents of age are 193,635; total respondents of religion, underlying disease and village health volunteer are 142,520.

Our analysis of quantitative survey findings and qualitative in-depth interviews produced three themes. First, the trend of vaccine acceptance, hesitance and refusal; second, trustworthy sources of information; and third, the impact of vaccine information and other factors on vaccine uptake.

### Theme 1: Trend of vaccine acceptance, hesitance and refusal

We defined vaccine acceptance as those who reported they want a vaccine; hesitance as those who were still indecisive to whether they will get a vaccine or not; and refusal as those who definitely rejected a vaccine.

**Fig 2:**
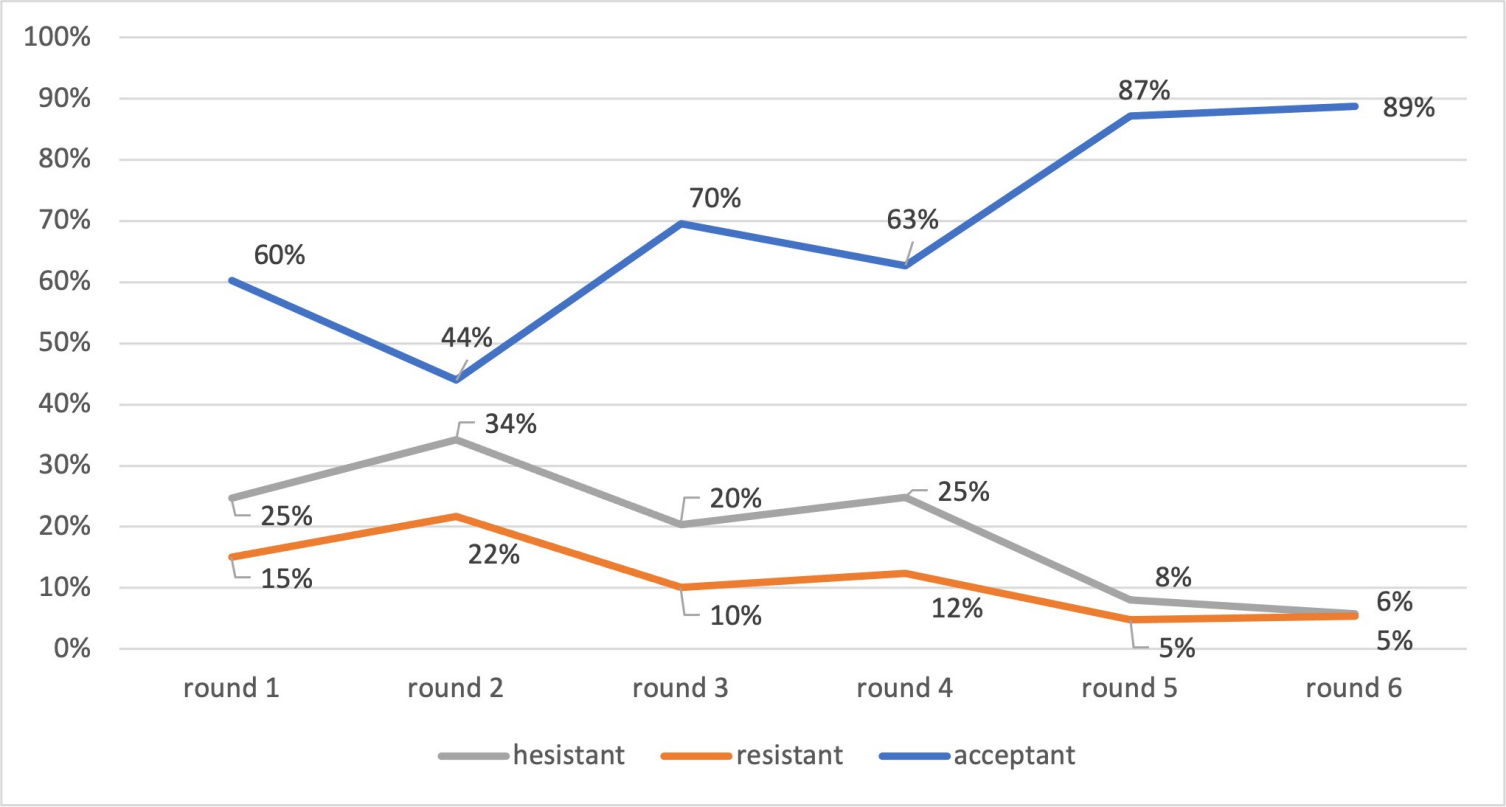
The trend of vaccine acceptance, hesitance and refusal.

The overall trend of vaccine acceptance increased over the six surveys. About 60% of all respondents accepted vaccines in March 2021 then it decreased to 44.0% in April 2021; it increased to 69.6% in May 2021 and peaked at 88.8% in August 2021. At the same time, 24.7% hesitancy in March 2021 decreased to 8.1% and 5.8% in July and August 2021. Similarly, the number of those who refused a vaccine decreased over the six-month period from 15.0% in March to 5.4% in August 2021. See Fig 2.

### Theme 2: Trustworthy sources of information

**Fig 3:**
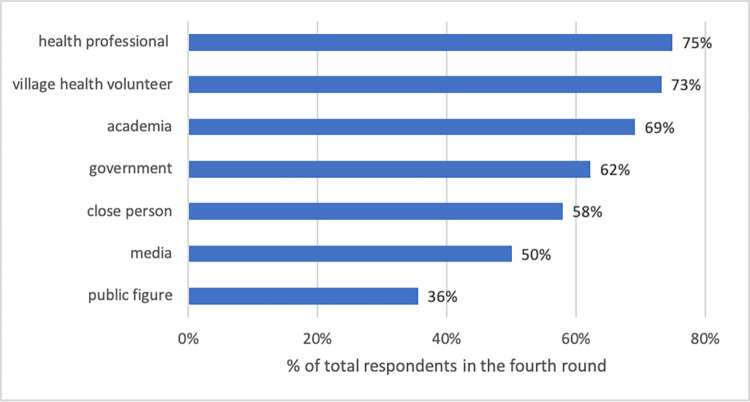
The proportion of respondents who trusted different sources of information. Note: n = 26,037.

From the survey, health professionals (74.9%), VHVs (73.2%), and academics (69.1%) were reported as the three most trustworthy sources of vaccine information. The government, family and friends, and the media were reported as less trustworthy accounting for 62.2%, 58.0% and 50.0% of respondents respectively. Public figures, such as celebrity or social media influencers, were seen as having the least trustworthy source of vaccine information (35.6%). See Fig 3.

From in-depth interviews, the respondents had different judgments subject to their ability to understand, appraise information they received and search for additional evidence to verify information they received. Interviewees who were frontline health staff or had higher education proactively sought and verified with additional evidence to confirm the reliability of the information they received, usually through reliable academic journals, or international media published by WHO or UNICEF. Non-health professional interviewees commonly received information from healthcare workers and media through various channels such as online media (Facebook, Twitter, Clubhouse application) and television. Older people or people who were not able to search additional information on their own often got information from television and trusted individuals such as their younger relatives or close friends. A few key informants relied on their trusted sources and ignored sources that they considered unreliable, while some sought multiple sources of information before making their decisions.

“*(I get my information) mainly from my children*. […] *My son told me not to go out if I am not vaccinated because there are several infected cases in our neighborhood*.” (*EPRN2*)

Some key informants reported that information from official sources such as the Centre for COVID-19 Situation Administration (CCSA) were inaccessible, unclear and unreliable. They noted that there was no clear evidence on vaccine safety and efficacy, and they were not in line with international sources of information. They noted that CCSA promoted only information to support government-provided vaccines (in this case Sinovac and AstraZeneca). These key informants suggested the authorities should have proactively communicated by providing factual, understandable, accessible, and responsive information via appropriate communication channels for each population group.

*“One thing is that the government sector information is very difficult to access*. *Only people who are really interested can reach it*. *[*…*] When I tried googling government information*, *I found nothing*. *It’s really hard to access them*. *[*…*] Moreover*, *the government websites are not user-friendly*. *It is difficult to search*.*”* (IRY1)

### Theme 3: Impact of vaccine information and other factors on vaccine uptake

The exposure to misinformation and ability to differentiate true and false information was assessed in all six rounds and only high exposure and general knowledge statements, which were not limited to the Thai context, were selected for analysis. Table 2 contains eight statements of which four are true and four are false. Details of other statements are shown in the Supplementary file, see S2 Table.

**Table 2:** The number and proportion of respondents who were exposed to and provided correct and incorrect answers to eight selected true and false statements.

No.	Statement	T/F	Number of respondents who exposed to statement (%)			
			Correct answer	Incorrect answer	Unsure	Total
1	Vaccines used in Thailand (Sinovac & Astrazeneca) have low efficacy in preventing severe illness and death	F	2,804 (16.0)	7,593 (43.4)	7,112 (40.6)	17,509 (100.0)
2	Sinovac vaccine causes permanent effect of stroke	F	2,461 (16.8)	4,912 (33.6)	7,248 (49.6)	14,621 (100.0)
3	If there are no side effects after vaccination, it means no or low level of immunity	F	2,696 (23.4)	5,087 (44.2)	3,737 (32.4)	11,520 (100.0)
4	If you have low immunization level after vaccination, it means no immunity	F	1,872 (21.5)	3,516 (40.3)	3,336 (38.2)	8,724 (100.0)
5	Side effects such as headache, fever, nausea are common symptoms after vaccination	T	26,794 (65.6)	1,476 (3.6)	12,590 (30.8)	40,860 (100.0)
6	Pregnant (gestational age more than 12 weeks) or breastfeeding woman can get the vaccine	T	12,397 (69.0)	825 (4.6)	4,754 (26.4)	17,976 (100.0)
7	People who are allergic to foods or have allergic rhinitis, can get the vaccine	T	11,900 (66.2)	1,097 (6.1)	4,988 (27.7)	17,985 (100.0)
8	If you have had a COVID-19 infection and are already cured, you should also get the vaccine	T	16,545 (87.8)	233 (1.2)	2,078 (11.0)	18,856 (100.0)

Note: False statement (F); True statement (T).

In Table 2, more than half of respondents have seen these eight statements. Those exposed to true statements were more likely to give correct answers compared to those who saw false statements.

In the surveys, we maintained certain key parameters for trend monitoring which can inform policy in all six rounds. There was an upward trend of confidence in COVID-19 vaccines in terms of safety, efficacy and that vaccination was important to them; although trust in government, healthcare providers and vaccine producers saw a less significant increasing trend. In the first round of the survey (March 2021), less than half of respondents agreed that the vaccines are safe (40.3%), vaccines are important for them (42.9%) and vaccines are effective (44.4%). Participants’ confidence on safety and efficacy rose to more than 60% in May 2021. In round 6 (August 2021), confidence in vaccines reached its peak with respondents saying that the vaccine was important for them (78.0%), effective (73.0%) and safe (71.7%).

**Fig 4:**
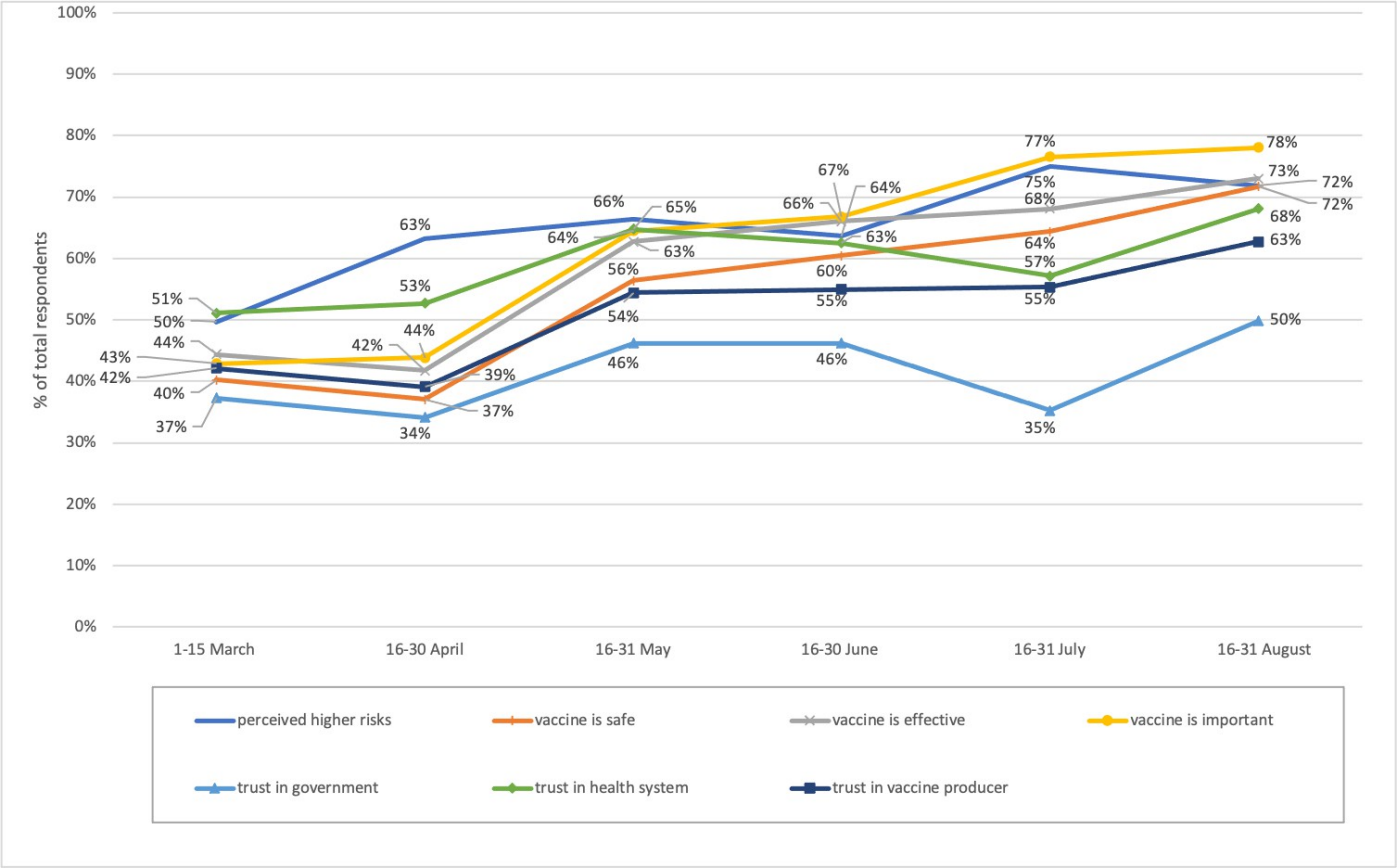
Trend of key parameters related to COVID-19 vaccine in the population.

In the first round of the survey (March 2021), more than half of participants trusted healthcare providers (51.1%) while around two-fifths trusted vaccine producers and the government (42.2% and 37.3% respectively). See Fig 4. However, trust in healthcare providers and the government increased slightly then dropped significantly in July by 5 to 10 percentage points. Nevertheless, in the last survey round in August 2021, most respondents trusted the health system (68.1%) followed by vaccine producers (62.7%). The government had the least trust, at less than 50%. The high level of perceived risk of COVID-19 infection increased from 49.7% in the first round of survey to 71.8% in the last round.

From the parameters generated from quantitative surveys, we conducted multivariable binary logistic regression analysis on the association between demographic characteristics and other factors on vaccine acceptance, categorized by accept, or hesitant that combined refusal. Tables 3 and 4 analyzed participants who had been exposed to eight selected true and false statements.

**Table 3:** The frequency of vaccine acceptance classified by various factors among respondents who were exposed to eight selected true and false statements.

Variables	Subgroup	Among those who exposed to each statement															
		Vaccines used in Thailand (Sinovac & Astrazeneca) have low efficacy in preventing severe illness and death (F)		Sinovac vaccine causes permanent effect of stroke (F)		If there are no side effects after vaccination, it means no or low level of immunity (F)		If you have low immunization level after vaccination, it means no immunity (F)		Side effects such as headache, fever, nausea are common symptoms after vaccination (T)		Pregnant (GA more than 12 weeks) or breastfeeding woman can get the vaccine (T)		People who are allergic to foods or have allergic rhinitis can get the vaccine (T)		If you have had COVID-19 infection and are already cured, you should also get the vaccine (T)	
		Hesitate/ Refuse	Accept	Hesitate/ Refuse	Accept	Hesitate/ Refuse	Accept	Hesitate/ Refuse	Accept	Hesitate/ Refuse	Accept	Hesitate/ Refuse	Accept	Hesitate/ Refuse	Accept	Hesitate/ Refuse	Accept
**Age (year)**	≤ 60	3,782 (32.3)	7,922 (67.7)	3,434 (34.5)	6,508 (65.5)	1,022 (10.9)	8,339 (89.1)	635 (9.3)	6,173 (90.7)	15,133 (55.5)	12,155 (44.5)	3,350 (28.8)	8,278 (71.2)	2,822 (34.3)	5,429 (65.7)	1,501 (9.6)	14,124 (90.4)
	> 60	722 (24.2)	2,265 (75.8)	661 (26.0)	1,886 (74.0)	328 (15.2)	1,831 (84.8)	231 (12.1)	1,685 (87.9)	3,689 (55.2)	2,998 (44.8)	661 (21.0)	2,483 (79.0)	600 (29.4)	1,440 (70.6)	430 (13.3)	2,798 (86.7)
**Gender**	male	855 (30.0)	2,000 (70.0)	795 (33.2)	1,599 (66.8)	301 (12.4)	2,120 (87.6)	254 (13.3)	1,657 (86.7)	3,039 (50.6)	2,968 (49.4)	894 (28.9)	2,195 (71.1)	810 (34.4)	1,545 (65.6)	434 (10.4)	3,745 (89.6)
	female	3,625 (30.8)	8,143 (69.2)	3,208 (32.7)	6,758 (67.3)	1,043 (11.6)	7,981 (88.4)	606 (9.0)	6,166 (91.0)	15,600 (56.3)	12,096 (43.7)	3,102 (26.7)	8,534 (73.3)	2,610 (33.0)	5,302 (67.0)	1,485 (10.2)	13,044 (89.8)
**Education level**	secondary school or lower	3,871 (33.3)	7,738 (66.7)	3,534 (35.2)	6,507 (64.8)	1,110 (14.8)	6,379 (85.2)	773 (11.1)	6,218 (88.9)	15,796 (57.8)	11,512 (42.2)	3,249 (28.7)	8,086 (71.3)	2,821 (35.2)	5,194 (64.8)	1,549 (13.3)	10,070 (86.7)
	university or higher	633 (20.5)	2,449 (79.5)	561 (22.9)	1,887 (77.1)	240 (6.0)	3,791 (94.0)	93 (5.4)	1,640 (94.6)	3,026 (45.4)	3,641 (54.6)	762 (22.2)	2,675 (77.8)	612 (26.8)	1,675 (73.2)	382 (5.3)	6,852 (94.7)
**Religion**	Buddhist	4,120 (29.8)	9,703 (70.2)	3,770 (32.0)	8,008 (68.0)	1,211 (11.3)	9,510 (88.7)	809 (10.0)	7,322 (90.0)	17.644 (54.8)	14,561 (45.2)	3,702 (26.5)	10,270 (73.5)	3,235 (32.6)	6,680 (67.4)	1,698 (9.8)	15,701 (90.2)
	Others^α^	384 (44.2)	484 (55.8)	325 (45.7)	386 (54.3)	139 (17.4)	660 (82.6)	57 (9.6)	536 (90.4)	1,158 (66.2)	592 (33.8)	309 (38.6)	491 (61.4)	198 (51.2)	189 (48.8)	233 (16.0)	1,221 (84.0)
**Occupation**	low risk^β^	3,563 (30.7)	8,056 (69.3)	3,258 (32.9)	6,644 (67.1)	1,020 (12.0)	7,518 (88.0)	695 (10.5)	5,916 (89.5)	14,641 (55.9)	11,540 (44.1)	3,185 (27.3)	8,486 (72.7)	2,640 (34.2)	5,077 (65.8)	1,447 (10.5)	12,356 (89.5)
	high risk^β^	941 (30.6)	2,131 (69.4)	837 (32.4)	1,750 (67.6)	303 (11.1)	2,652 (88.9)	171 (8.1)	1,942 (91.9)	4,181 (53.6)	3,613 (46.4)	826 (26.6)	2,275 (73.4)	793 (30.7)	1,792 (69.3)	484 (9.6)	4,566 (90.4)
**Region**	low risk^δ^	N/A	N/A	N/A	N/A	216 (24.1)	681 (75.9)	N/A	N/A	N/A	N/A	N/A	N/A	2,222 (35.3)	4,065 (64.7)	308 (21.6)	1,115 (78.4)
	high risk^δ^	4,504 (30.7)	10,187 (69.3)	4,095 (32.8)	8,394 (67.2)	1,134 (10.7)	9,489 (89.3)	866 (9.9)	7,858 (90.1)	18,822 (55.4)	15,153 (44.6)	4,011 (27.2)	10,761 (72.8)	1,211 (30.2)	2,804 (69.8)	1,623 (9.3)	15.807 (90.7)
**Underlying disease**	low risk^γ^	3,088 (30.5)	7,047 (69.5)	2,771 (32.3)	5,813 (67.7)	859 (10.5)	7,354 (89.5)	590 (9.5)	5,631 (90.5)	13,807 (54.5)	11,518 (45.5)	2,768 (26.9)	7,525 (73.1)	2,309 (31.6)	4,995 (68.4)	1,249 (9.1)	12,493 (90.9)
	high risk^γ^	1,416 (31.1)	3,140 (68.9)	1,324 (33.9)	2,581 (66.1)	491 (14.9)	2,816 (85.1)	276 (11.0)	2,227 (89.0)	5,015 (58.0)	3,635 (42.0)	1,243 (27.8)	3,236 (72.2)	1,124 (37.5)	1,1874 (62.5)	682 (13.3)	4,429 (86.7)
**Health volunteer status**	no	1,038 (29.2)	2,514 (70.8)	974 (32.0)	2,068 (68.0)	533 (11.5)	4,104 (88.5)	394 (17.2)	1,904 (82.8)	4,114 (52.0)	3,792 (48.0)	1,143 (29.6)	2,725 (70.4)	1,135 (36.2)	2,000 (63.8)	735 (9.3)	7,191 (90.7)
	yes	3,466 (31.1)	7,673 (68.9)	3,121 (33.0)	6,326 (67.0)	817 (11.9)	6,066 (88.1)	472 (7.4)	5,954 (92.6)	14,708 (56.4)	11,361 (43.6)	2,868 (26.3)	8,036 (73.7)	2,298 (32.1)	4,869 (67.9)	1,196 (11.0)	9,731 (89.0)
**Ability to differentiate information**	Incorrect/ unsure	4,096 (32.6)	8,459 (67.4)	3,739 (35.3)	6,854 (64.7)	1,130 (12.8)	7,694 (87.2)	734 (10.7)	6,118 (89.3)	7,528 (60.4)	4,944 (39.6)	1,622 (34.3)	3,122 (65.7)	1,760 (45.9)	2,075 (54.1)	478 (20.7)	1,833 (79.3)
	correct	408 (19.1)	1,728 (80.9)	356 (18.8)	1,540 (81.2)	220 (8.2)	2,475 (91.8)	132 (7.1)	1,740 (92.9)	11,294 (52.5)	10,209 (47.5)	2,378 (23.7)	7,639 (76.3)	1,673 (25.9)	4,794 (74.1)	1,453 (8.8)	15,089 (91.2)
**Risk perception**	low to moderate	3,629 (37.6)	6,012 (62.4)	3,296 (40.2)	4,914 (59.8)	994 (16.1)	5,182 (83.9)	674 (14.6)	3,953 (85.4)	15,229 (64.3)	8,464 (35.7)	3,213 (34.3)	6,155 (65.7)	2,948 (43.6)	3,813 (56.4)	1,440 (14.6)	8,456 (85.4)
	high	875 (17.3)	4,175 (82.7)	799 (18.7)	3,480 (81.3)	356 (6.7)	4,988 (93.3)	192 (4.7)	3,905 (95.3)	3,593 (34.9)	6,689 (65.1)	798 (14.8)	4,606 (85.2)	485 (13.7)	3,056 (86.3)	491 (5.5)	8,466 (94.5)
**Vaccine safety**	low to moderate	3,304 (45.5)	3,956 (54.5)	3,057 (47.6)	3,364 (52.4)	770 (20.1)	3,065 (79.9)	483 (18.6)	2,111 (81.4)	15,714 (69.4)	6,915 (30.6)	2,848 (44.0)	3,627 (56.0)	2,386 (55.0)	1,956 (45.0)	1,154 (18.9)	4,965 (81.1)
	high	1,200 (16.2)	6,231 (83.8)	1,038 (17.1)	5,030 (82.9)	580 (7.6)	7,105 (92.4)	383 (6.3)	5,747 (93.7)	3,108 (27.4)	8,238 (72.6)	1,163 (14.0)	7,134 (86.0)	1,047 (17.6)	4,913 (82.4)	777 (6.1)	11,957 (93.9)
**Vaccine effectiveness**	low to moderate	2,872 (46.6)	3,296 (53.4)	2,667 (48.4)	2,839 (51.6)	665 (20.7)	2,551 (79.3)	420 (17.6)	1,972 (82.4)	14,517 (70.0)	6,215 (30.0)	2,501 (44.8)	3,079 (55.2)	2,080 (57.1)	1,565 (42.9)	980 (18.7)	4,256 (81.3)
	high	1,632 (19.2)	6,891 (80.8)	1,428 (20.4)	5,555 (79.6)	685 (8.3)	7,619 (91.7)	444 (7.0)	5,886 (93.0)	4.305 (32.5)	8,938 (67.5)	1,510 (16.4)	7,682 (83.6)	1,353 (20.3)	5,304 (79.7)	951 (7.0)	12,666 (93.0)
**Vaccine importance**	low to moderate	2,976 (50.9)	2,875 (49.1)	2,749 (52.4)	2,495 (47.6)	695 (27.2)	1,858 (72.8)	420 (21.1)	1,573 (78.9)	14.819 (73.3)	5,400 (26.7)	2,625 (48.6)	2,772 (51.4)	2,272 (60.8)	1,467 (39.2)	1,044 (27.2)	2,794 (72.8)
	high	1,528 (17.3)	7,312 (82.7)	1,346 (18.6)	5,899 (81.4)	655 (7.3)	8,312 (92.7)	446 (6.6)	6,285 (93.4)	4,003 (29.1)	9,753 (70.9)	1,386 (14.8)	7,989 (85.2)	1,161 (17.7)	5,402 (82.3)	887 (5.9)	14,128 (94.1)
**Trust in government**	low to moderate	3,394 (38.8)	5,344 (61.2)	3,124 (41.2)	4,463 (58.8)	859 (12.4)	6,061 (87.6)	537 (11.5)	4,141 (88.5)	15,028 (65.1)	8,056 (34.9)	2,917 (37.3)	4,897 (62.7)	2,501 (46.1)	2,923 (53.9)	1,327 (11.2)	10,538 (88.8)
	high	1,110 (18.7)	4,843 (81.3)	971 (19.8)	3,931 (80.2)	491 (10.7)	4,109 (89.3)	329 (8.1)	3,717 (91.9)	3,794 (34.8)	7,097 (65.2)	1,094 (15.7)	5,864 (84.3)	932 (19.1)	3,946 (80.9)	604 (8.6)	6,384 (91.4)
**Trust in healthcare provider**	low to moderate	2,481 (41.3)	3,530 (58.7)	2,336 (43.7)	3,009 (56.3)	666 (14.4)	3,960 (85.6)	396 (13.1)	2,634 (86.9)	11,045 (66.6)	5,539 (33.4)	2,232 (40.8)	3,238 (59.2)	1,983 (51.0)	1,902 (49.0)	993 (12.9)	6,705 (87.1)
	high	2,023 (23.3)	6,657 (76.7)	1,759 (24.6)	5,385 (75.4)	684 (9.9)	6,210 (90.1)	470 (8.3)	5,224 (91.7)	7,777 (44.7)	9,614 (55.3)	1,779 (19.1)	7,523 (80.9)	1,450 (22.6)	4,967 (77.4)	938 (8.4)	10,217 (91.6)
**Trust in vaccine producer**	low to moderate	3,185 (42.5)	4,313 (57.5)	2,941 (44.7)	3,638 (55.3)	769 (16.0)	4,033 (84.0)	465 (13.4)	3,008 (86.6)	14,572 (67.7)	6,968 (32.3)	2,753 (41.4)	3,898 (58.6)	2,356 (50.8)	2,279 (49.2)	1,194 (15.0)	6,791 (85.0)
	high	1,319 (18.3)	5,874 (81.7)	1,154 (19.5)	4,756 (80.5)	581 (8.7)	6,137 (91.3)	401 (7.6)	4,858 (92.4)	4,250 (34.2)	8,185 (65.8)	1,258 (15.5)	6,863 (84.5)	1,077 (19.0)	4,590 (81.0)	737 (6.8)	10,131 (93.2)
**Total**		**4,504 (30.7)**	**10,187 (69.3)**	**4,095 (32.8)**	**8,394 (67.2)**	**1,350 (11.7)**	**10,170 (88.3)**	**866 (9.9)**	**7,858 (90.1)**	**18,882 (55.4)**	**15,153 (44.6)**	**4,011 (27.2)**	**10,761 (72.8)**	**3,433 (33.3)**	**6,869 (66.7)**	**1,931 (10.2)**	**16,922 (89.8)**

Note

^α^ Other religions are Muslim, Christian and others

^β^ Low risk occupations are agricultural worker, unemployed, student, employee in public and private sectors and high risk occupations are freelance, daily worker, driver, business owner and health professional

^δ^ low risk area is minimum-risk provinces and high risk areas are extreme high-risk provinces, High-risk provinces and Moderate-risk provinces

^γ^ Low risk underlying diseases are healthy and other diseases and high risk underlying diseases are seven priority diseases.

False statements (F); True statements (T); Gestational age (GA); N/A is not applicable due to no data or perfect collinearity.

**Table 4:** The multivariable binary *logistic regression* analysis of association between various factors and vaccine acceptance among respondents who were exposed to eight selected true and false statements.

Variables	Subgroup	Among those who exposed to each statement															
		Vaccines used in Thailand (Sinovac & Astrazeneca) have low efficacy in preventing severe illness and death (F)		Sinovac vaccine causes permanent effect of stroke (F)		If there are no side effects after vaccination, it means no or low level of immunity (F)		If you have low immunization level after vaccination, it means no immunity (F)		Side effects such as headache, fever, nausea are common symptoms after vaccination (T)		Pregnant (GA more than 12 weeks) or breastfeeding woman can get the vaccine (T)		People who are allergic to foods or have allergic rhinitis can get the vaccine (T)		If you have had COVID-19 infection and are already cured, you should also get the vaccine (T)	
		OR (95%CI)	P-value	OR (95%CI)	P-value	OR (95%CI)	P-value	OR (95%CI)	P-value	OR (95%CI)	P-value	OR (95%CI)	P-value	OR (95%CI)	P-value	OR (95%CI)	P-value
**Age (year)**	≤ 60	Reference		Reference		Reference		Reference		Reference		Reference		Reference		Reference	
	> 60	1.6 (1.5–1.8)	<0.001***	1.6 (1.5–1.8)	<0.001***	0.8 (0.7–1.0)	0.016*	0.9 (0.8-.1.1)	0.209	1.1 (1.0–1.1)	0.029*	1.6 (1.4–1.8)	<0.001***	1.3 (1.2–1.5)	<0.001***	0.8 (0.7–1.0)	0.006**
**Gender**	male	Reference		Reference		Reference		Reference		Reference		Reference		Reference		Reference	
	female	1.0 (0.9–1.1)	0.747	1.0 (0.9–1.2)	0.401	1.0 (0.8–1.1)	0.702	1.1 (0.9–1.3)	0.423	0.8 (0.8–0.9)	<0.001***	1.1 (1.0–1.2)	0.125	1.0 (0.9–1.1)	0.403	1.0 (0.8–1.1)	0.510
**Education level**	secondary school or lower	Reference		Reference		Reference		Reference		Reference		Reference		Reference		Reference	
	university or higher	2.3 (2.1–2.2)	<0.001***	2.2 (1.9–2.4)	<0.001***	4.1 (3.5–4.9)	<0.001***	3.4 (2.7–4.3)	<0.001***	1.7 (1.6–1.8)	<0.001***	1.8 (1.6–2.0)	<0.001***	1.6 (1.4–1.8)	<0.001***	3.7 (3.2–4.3)	<0.001***
**Religion**	Buddhist	Reference		Reference		Reference		Reference		Reference		Reference		Reference		Reference	
	Others^α^	0.5 (0.5–0.6)	<0.001***	0.5 (0.5–0.6)	<0.001***	0.5 (0.4–0.6)	<0.001***	1.0 (0.8–1.3)	0.977	0.6 (0.5–0.7)	<0.001***	0.6 (0.5–0.7)	<0.001***	0.4 (0.3–0.5)	<0.001***	0.4 (0.4–0.5)	<0.001***
**Occupation**	low risk^β^	Reference		Reference		Reference		Reference		Reference		Reference		Reference		Reference	
	high risk^β^	1.0 (0.9–1.1)	0.677	1.0 (1.0–1.1)	0.355	1.1 (0.9–1.2)	0.396	1.2 (1.0–1.5)	0.024*	1.1 (1.0–1.2)	<0.001***	1.1 (1.0–1.2)	0.289	1.1 (1.0–1.3)	0.011*	1.1 (1.0–1.2)	0.115
**Region**	low risk^β^	Reference		Reference		Reference		Reference		Reference		Reference		Reference		Reference	
	high risk^β^	N/A	N/A	N/A	N/A	3.0 (2.5–3.6)	<0.001***	N/A	N/A	N/A	N/A	N/A	N/A	1.4 (1.3–1.5)	<0.001***	2.9 (2.5–3.4)	<0.001***
**Underlying disease**	low risk^γ^	Reference		Reference		Reference		Reference		Reference		Reference		Reference		Reference	
	high risk^γ^	0.9 (0.8–1.0)	0.024*	0.9 (0.8–0.9)	0.001**	0.7 (0.6–0.8)	<0.001***	0.8 (0.7–0.9)	0.009**	0.9 (0.8–0.9)	<0.001***	0.9 (0.8–1.0)	0.002**	0.7 (0.6–0.8)	<0.001***	0.7 (0.6–0.8)	<0.001***
**Health volunteer status**	no	Reference		Reference		Reference		Reference		Reference		Reference		Reference		Reference	
	yes	1.3 (1.1–1.4)	<0.001***	1.2 (1.1–1.4)	<0.001***	2.4 (2.1–2.8)	<0.001***	3.4 (2.9–4.0)	<0.001***	1.1 (1.0–1.1)	0.144	1.5 (1.3–1.6)	<0.001***	1.6 (1.5–1.8)	<0.001***	2.0 (1.8–2.3)	<0.001***
**Ability to differentiate information**	Incorrect/unsure	Reference		Reference		Reference		Reference		Reference		Reference		Reference		Reference	
	correct	1.9 (1.7–2.1)	<0.001***	2.2 (2.0–2.5)	<0.001***	1.2 (1.0–1.5)	0.012*	1.5 (1.2–1.8)	<0.001***	1.3 (1.3–1.4)	<0.001***	2.3 (2.1–2.5)	<0.001***	2.4 (2.2–2.6)	<0.001***	2.4 (2.1–2.7)	<0.001***
**Risk perception**	low to moderate	Reference		Reference		Reference		Reference		Reference		Reference		Reference		Reference	
	high	2.9 (2.6–3.1)	<0.001***	2.9 (2.7–3.2)	<0.001***	2.7 (2.4–3.1)	<0.001***	3.5 (3.0–4.2)	<0.001***	3.3 (3.2–3.5)	<0.001***	3.1 (2.8–3.3)	<0.001***	4.7 (4.2–5.3)	<0.001***	2.6 (2.9–3.3)	<0.001***
**Vaccine safety**	low to moderate	Reference		Reference		Reference		Reference		Reference		Reference		Reference		Reference	
	high	2.3 (2.0–2.6)	<0.001***	2.4 (2.1–2.7)	<0.001***	1.4 (1.1–1.7)	0.005**	2.3 (1.7–3.0)	<0.001***	2.4 (2.2–2.6)	<0.001***	2.3 (2.0–2.6)	<0.001***	2.1 (1.8–2.4)	<0.001***	1.5 (1.2–1.8)	<0.001***
**Vaccine effectiveness**	low to moderate	Reference		Reference		Reference		Reference		Reference		Reference		Reference		Reference	
	high	0.7 (0.6–0.9)	<0.001***	0.7 (0.6–0.9)	<0.001***	0.7 (0.6–0.9)	0.014*	0.9 (0.6–1.1)	0.284	0.9 (0.9–1.0)	0.119	0.7 (0.6–0.8)	<0.001***	0.9 (0.7–1.0)	0.097	0.8 (0.6–0.9)	0.003**
**Vaccine importance**	low to moderate	Reference		Reference		Reference		Reference		Reference		Reference		Reference		Reference	
	high	3.4 (3.0–3.9)	<0.001***	3.3 (2.8–3.8)	<0.001***	4.6 (3.7–5.7)	<0.001***	2.3 (1.8–3.0)	<0.001***	3.8 (3.6–4.1)	<0.001***	4.0 (3.4–4.6)	<0.001***	4.6 (3.9–5.5)	<0.001***	5.1 (4.3–6.1)	<0.001***
**Trust in government**	low to moderate	Reference		Reference		Reference		Reference		Reference		Reference		Reference		Reference	
	high	1.5 (1.3–1.7)	<0.001***	1.5 (1.3–1.7)	<0.001***	0.6 (0.5–0.8)	<0.001***	1.1 (0.9–1.4)	0.388	1.8 (1.6–1.9)	<0.001***	1.4 (1.2–1.6)	<0.001***	1.4 (1.2–1.6)	<0.001***	0.7 (0.6–0.8)	<0.001***
**Trust in healthcare provider**	low to moderate	Reference		Reference		Reference		Reference		Reference			Reference	Reference		Reference	
	high	0.9 (0.8–1.0)	0.015*	0.9 (0.8–1.0)	0.057	1.1 (0.9–1.4)	0.212	1.1 (0.9–1.4)	0.449	0.8 (0.7–0.8)	<0.001***	1.0 (0.9–1.1)	0.742	1.3 (1.1–1.5)	<0.001***	1.1 (0.9–1.2)	0.295
**Trust in vaccine producer**	low to moderate	Reference		Reference		Reference		Reference		Reference		Reference		Reference		Reference	
	high	2.8 (2.4–3.1)	<0.001***	2.8 (2.5–3.2)	<0.001***	2.8 (2.3–3.3)	<0.001***	1.9 (1.5–2.4)	<0.001***	3.2 (2.9–3.4)	<0.001***	3.0 (2.7–3.5)	<0.001***	2.8 (2.4–3.2)	<0.001***	3.1 (2.6–3.5)	<0.001***

Note

^α^ Other religions are Muslim, Christian and others

^β^ Low risk occupations are agricultural worker, unemployed, student, employee in public and private sectors and high risk occupations are freelance, daily worker, driver, business owner and health professional; ^δ^ low risk area is minimum-risk provinces and high risk areas are extreme high-risk provinces, High-risk provinces and Moderate-risk provinces

^γ^ Low risk underlying diseases are healthy and other diseases and high risk underlying diseases are seven priority diseases.

False statements (F); True statements (T); Gestational age (GA); N/A is not applicable due to no data or perfect collinearity.

Odds ratio (OR) more than one means higher probability to accept vaccine while odds ratio less than one means lower probability to accept vaccine; *statistically significant at 95% confident level (P-value<0.05); **statistically significant at 95% confident level (P-value<0.01)

***statistically significant at 95% confident level (P-value<0.001).

Analysis suggested that education, health status, area of residence, risk perception, perceived importance and safety of vaccine, trust in vaccine manufacturers, and ability to differentiate true and false statements significantly correlated with willingness to vaccinate.

In Table 3, respondents who had the ability to differentiate true and false information were 1.2–2.4 times more likely to accept vaccine (P-value<0.001–0.012). Respondents who had secondary or higher levels of education tended accept vaccination more than those with completed primary education (Adjusted odds ratio (AOR) = 1.6–4.1; P-value<0.001). Additionally, those who perceived a higher risk of infection (AOR = 2.6–4.7; P-value < 0.001), and perceived vaccine safety (AOR = 1.4–2.4; P-value<0.001–0.005) and the importance of vaccination (AOR = 2.3–5.1; P-value<0.001) were more likely to accept vaccination.

Respondents who had higher risks of infection such as chronic diseases (AOR = 0.7–0.9; P-value<0.001–0.024) were less likely to accept vaccination than healthy people, whereas those who lived in high-risk areas (high outbreak numbers) tended to have higher vaccine acceptance than those lived in low-risk areas (AOR = 1.4–3.0; P-value<0.001). Participants who trusted the vaccine producer were also more likely to get vaccinated (AOR = 1.9–3.2; P-value<0.001).

From interviews with key informants, there were various reasons for accepting, hesitating or refusing vaccination. Our multivariable binary logistic regression analysis clearly showed risk perception was one of the key determinants, followed by areas in which they lived, health status, or their work status or lifestyle, which exposed them to infection. This was also confirmation from in-depth interviews with, for example, interviewees who lived in areas with high outbreaks and those with jobs that exposed them to many people who have asymptomatic infections, were more likely to get vaccinated. Some interviewees with chronic diseases decided to vaccinate due to their personal risks but some were still concerned about the side effects and became hesitant. Those who were reluctant to be vaccinated usually perceived a low risk of infection and felt that they could protect themselves from infection.

*“I really have not met anyone but the delivery people*. *And I have no reason to worry about risk*, *because I do not have to go out and meet people*. *So*, *I think*, *it is OK that I can wait (and see the vaccine)*.*”* (IRY2)

Interviewees also described different degrees of vaccine confidence. Some recommended that the risk of side effects (which is extremely low) should be a trade-off with the benefits of the vaccine (which meant minimizing risk of mortality once infected). The benefits of the vaccine were perceived differently across individuals, for instance, some decided to wait for vaccines that had higher efficacy to prevent infection, whereas some individuals thought that benefits gained from reduced morbidity and mortality was more than enough. They also considered the importance of being vaccinated in terms of their own protection and to protect others such as family members. This means that if they are not infected, they will not pass infections to others in the family. Although the vaccine acceptance rate was generally increasing, some interviewees still refused the vaccine because they wanted to wait for the most effective vaccine; however, some finally accepted the vaccines provided by the government as their preferred vaccines. At that time they were viral vector AstraZeneca and inactivated Sinovac, as mRNA such as Pfizer and Moderna were not available.

*“I think all vaccines can prevent severity of infection and minimize mortality*. *Although*, *some people said that Sinovac is imperfect*, *I still believe that it can prevent morbidity so it is enough to avoid overwhelming the health system … Moreover*, *I am afraid that I can be a virus carrier to my parents and grandmother who are both at risk of severity if infected*.*”* (ORY3)

Finally, religious belief seems to have limited effects on vaccine acceptance. Though Halal is one of the concerns among the Muslim community, the Chulalongkorn University Halal Science Center has proved and announced that the COVID-19 vaccine is Halal and this was further endorsed by The Central Islamic Council of Thailand. Misconceptions about vaccines and other preventive practices spread among some groups of Muslims. However, Muslim leaders reassured that having a COVID-19 vaccination does not conflict with Islamic principles. Many Muslim populations, especially in deep south provinces, also received information from the media and people in other Islamic countries owing to the similarity in religion and language. Muslim leaders and the believers suggested that the government should appoint an influential ambassador who people in the community respect and trust, such as imams, Muslim medical personnel, Sheikhul Islam Office and the Provincial Islamic Committees, to help disseminate accurate information in local languages and work with local health authorities.

*“There was a message about the vaccine and the side effects they received from Malaysia*. *I saw it too*. *[*…*] First of all*, *(it is popular because) we share the same religion*, *as most Malays*, *especially those who live near the border*, *are Muslims like us*. *And second*, *the language*, *which is very similar*. *It is an understandable language across the border*.*”* (MSY1)

## Discussion

The infodemic has exploded significantly, especially the novelty nature of the COVID-19 vaccine makes people feel uncertain about safety and efficacy as it was licensed as Emergency Use Authorization by the Thai Food and Drug Administration to combat the pandemic. The media was flooded with a mix of accurate and inaccurate information about vaccines that influenced people’s decision to vaccinate. Our study shows that levels of willingness to vaccinate are dynamic, and influenced by the following factors and context.

Levels of intention to vaccinate were low in first and second round of the surveys (March—April 2021) and then increased from May to the last round. Vaccine acceptance rates in our study are lower than a survey conducted by the University of Maryland and Meta Platforms, Inc., which showed average vaccine acceptance at more than 70% since April and increasing continuously since the start of the third wave [15]. However, that study was conducted online, and only those who can access the Internet, especially in urban areas (mostly with high number of daily COVID-19 cases), would dominate the survey findings even the non-response weighted. Vaccine hesitancy in Thailand is quite different from Western countries, which have active campaigns by anti-vax groups that completely reject vaccines [4] because the reluctant group in Thailand is larger than those who refuse. There are some Antivax campaigners in Thailand although they are not very active, and people have more trust in authentic sources of healthcare information. The undecided group (those saying they were unsure) was easier to convince to change their mind when grave pictures of cases and deaths caused by the pandemic emerged in the third quarter of 2021. There is a downward trend of the proportion of respondents who are hesitant or indecisive to vaccinate. Various factors such as perceived risk (having underlying chronic diseases, living in high-risk areas and having high-risk occupations) and improved confidence in the vaccine from updated information are among other factors influencing the change of their decisions.

Susceptibility to misinformation correlates with a lower rate of vaccine acceptance. A study in Korea shows that information avoidance, rather than information seeking, is a positive outcome of misinformation exposure [28]. Further, a one-unit increase of susceptibility to misinformation is associated with a 23% and 28% decrease of probability to vaccinate and recommending to others [1]. In Thailand during April, at the beginning of vaccination drive for frontline health workers, information about vaccine side effects spread rapidly inside and outside the country. For example, that Sinovac induces permanent strokes [29] or that AstraZeneca induces venous thrombosis [30]. This misinformation resulted in decreasing rates of vaccine acceptance.

**Fig 5:**
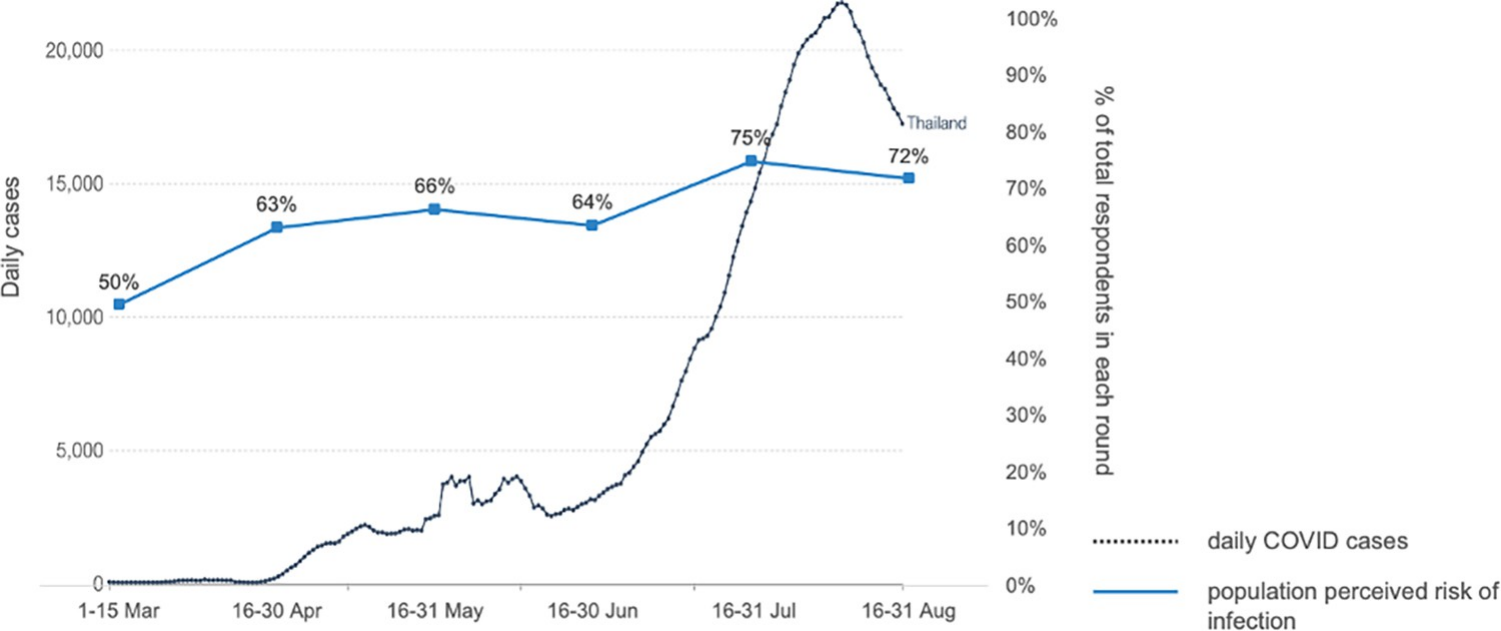
Trend of risk perception and daily cases of COVID-19 in Thailand between March and August 2021.

With reference to the five Cs model, confidence refers to people confidence on vaccine safety, efficacy and the importance of vaccination, while complacency refers to perceived risks [3]. Our study shows that the importance of vaccination, vaccine safety and risk perception are remarkably associated with the intention to vaccinate. The perceived risk of infection increased in line with the very large daily reported cases due to the Delta strain in the third quarter of 2021 [11], leading to higher number of vaccine acceptors. See Fig 5. At the beginning of the vaccine rollout campaign, people were concerned about vaccine safety so they were either hesitant or refused vaccines, but they perceived that evidence on the benefits from reduced mortality outweighed the risks of side effects [31]. Government and experts’ communication is very clear that vaccines do no prevent infection; even after vaccination people must maintain high coverage of facemasks and have good hand hygiene. But vaccines can minimize mortality among those who are infected after a full dose of vaccination. Further, the emergence of the third and fourth waves from Delta that almost overwhelmed the health system induced positive attitudes towards the importance of vaccinations and shaped citizens’ decisions towards acceptance.

The notion of waiting for better vaccines, such as mRNA, is confirmed in a study from the University of Maryland and Meta Platforms, Inc. [15]. Social media deliberately devalued vaccines managed by the Ministry of Public Health (inactivated Sinovac and viral vector AstraZeneca) as being of low efficacy, while advocating mRNA vaccines such as Pfizer or Moderna, which stimulated demand of alternative vaccines [32]. Accordingly, public authorities advocated with counteracting messages that, “the best vaccine is any vaccine available today” and ensured that the vaccines used in Thailand had efficacy to prevent mortality and morbidity [33]. This is an example of vaccine discourse influencing vaccination uptake.

As different vaccine arrived at different phases of the pandemic, it became inevitable to introduce heterologous vaccine schedules, so-called “mix and match”, such as two doses of Sinovac followed by AstraZeneca; or two doses of AstraZeneca followed by Pfizer. Media reported that people were unhappy with the Ministry’s mixed vaccine formula, citing safety reasons [34], despite Thailand’s clinical studies which proved increased immunogenicity and enhanced vaccine effectiveness, which were among key references used by WHO’s Strategic Advisory Group of Experts on Immunization in producing the interim recommendations for heterologous COVID-19 vaccine schedules [35].

Limited vaccine supplies at the beginning of the rollout were another barrier [36] which presented not only Thailand but all other low- and middle-income countries [37]. Affordability by the population was not a problem as all public vaccines were free for everyone including non-Thai population. Neither was accessibility an issue as Thailand has a strong health service infrastructure to deliver vaccinations. The problem of vaccine supply was finally resolved at the end of 2021 with increased vaccine production and importation [34].

Our concerns are that vulnerable groups such as low-educated people are more hesitant about or reject the vaccination. Findings from this study confirm scoping reviews which report that lower educational attainment is linked with poor vaccine uptake [6,7]. In this study, different religious beliefs did not significantly influence the intention to vaccinate as Muslim leaders were proactive in communicating with their communities. For example, they used scientific proof of the Halal status of COVID-19 vaccines [38] to help minimize the antivax movement in their communities, and the affirmation of Muslim leaders that the COVID-19 vaccine does not conflict with Islamic principles supported community choice to have the vaccine.

The public’s trust in vaccine quality and manufacturer found in this study confirms prior studies that trust supports vaccine uptake [4,39]. The trust in vaccine producers means trust in the quality of vaccine product [40], which correlates with vaccine acceptance. Although trust in the government is not significantly related to vaccine acceptance, trust in government is lower than trust in other organizations which also influences the trust in public communication as mentioned by some key informants compared to information from health professionals, academia and VHVs.

Several limitations are identified. Firstly, the survey is conducted through the VHV network; hence respondents are mostly VHVs and female which is not representative of the general population, and VHVs may have positive bias in favor of the vaccine. We realized this challenge and recruited more participants from other online channels to balance the overall samples; however, the samples are too small to rectify this weakness. Secondly, the six rounds of online surveys and self-administered questionnaires may introduce social-desirability bias; this means a tendency to underreport socially undesirable attitudes and behaviors and to over report more desirable attributes [41]. Thirdly, the COVID-19 situation, does not allow face-to-face interviews with key informants. This may affect the accuracy of information gathered, as researchers cannot observe body language or trigger frank interactions. Finally, key informants for in-depth interviews were recruited via online announcements so most of them are well connected to online media and the majority are female.

## Conclusion

The rollout of the COVID-19 vaccine is notably threatened by misinformation, other factors and the context during vaccine rollout, although we observed an upward trend of vaccine acceptance. The ability to distinguish between true and false statements determines vaccine acceptance. In addition, respondents who have a lower education or chronic diseases, those who perceived a lower risk of infection, are not confident in vaccine safety and importance, and distrust vaccine producers are either hesitant or refuse the vaccine. While combating misinformation, government campaign message could include that the benefits of the vaccine outweigh its side effects; that having the vaccine minimizes illness severity and risk of mortality but cannot prevent infection; and that fewer numbers of severe cases help to protect the overwhelmed health system. Actors such as VHVs and health personnel are key communicators and change agents for vaccine acceptance in the population. The transparent investigation of all adverse events following immunization and clear public communication based on available evidence are essential to gain citizens’ trust and ensure vaccine acceptance. Finally, regular monitoring of misinformation is important, supported by fact checking, timely legal actions and specific myth and misinformation debunking communication.

## Supporting information

S1 Table The characteristics of interviewees. (DOCX)

S2 Table The number and proportion of respondents who were exposed to all true and false statements from six round of surveys. (DOCX)

S3 Table Questionnaire. (DOCX)
