# Supplementary material for: The impact of vaccine information and other factors on COVID-19 vaccine acceptance in the Thai population
Source: PLoS One. 2023 Mar 7;18(3):e0276238. doi: 10.1371/journal.pone.0276238 (PMC9990931; doi:10.1371/journal.pone.0276238)
Supplement: S1 Table — (DOCX) [file pone.0276238.s001.docx]

**S1 Table. The characteristics of interviewees**

| **Group** | **Risk area**^α^ | **Sex** | **Code of Participants** |
| --- | --- | --- | --- |
|  |  |  |  |
| Health care workers | Extreme high to high-risk provinces | Male | HRY1 |
|  |  | Female | HRY2, HRN3 |
|  | Moderate to minimum-risk provinces | Female | HGN4, HGN5 |
|  |  | Male | HGN6 |
| People with NCDs:  aged of 18-59 years old | Extreme high to high-risk provinces | Female | PRY1, PRN2 |
|  | Moderate to minimum-risk provinces | Female | PGY3 PGN4 |
| People with NCDs:  aged over 60 years old | Extreme high to high-risk provinces | Female | EPRY1, EPRN2 |
|  | Moderate to minimum-risk provinces | Male | EPGY3 |
|  |  | Female | EPGN4 |
| Low-risk occupations^β^ | Extreme high to high-risk provinces | Female | IRY1, IRY2, IRN3, IRN4 |
|  | Moderate to minimum-risk provinces | Female | IGY5, IGY6, IGN7, IGN8 |
| High risk occupations^β^ | Extreme high to high-risk provinces | Female | ORY1, ORY2, ORN4, ORN5 |
|  |  | Male | ORY3 |
|  | Moderate to minimum-risk provinces | Male | OGN6 |
|  |  | Female | OGN7 |
| Muslims: believers | Deep South^δ^ | Female | MSY1 |
|  |  | Male | MSN2 |
|  | Other provinces | Male | MBY3, MBN4 |
| Muslims: religious leaders | Deep South^δ^ | Male | MLS1, MLS2 |
|  | Other provinces | Male | MLB3 |

Note: Non-communicable chronic diseases (NCDs)

^α^Based on the announcements of the government which ranged from extreme high-risk provinces (highest number of new cases, with full restrictions) to minimum-risk provinces (lowest number of new cases, with least restrictions) [12]

^β^Low-risk occupations are those who can work remotely while high-risk occupations are those who cannot work remotely

^δ^Pattani, Yala and Narathiwat
