# Supplementary material for: The impact of vaccine information and other factors on COVID-19 vaccine acceptance in the Thai population
Source: PLoS One. 2023 Mar 7;18(3):e0276238. doi: 10.1371/journal.pone.0276238 (PMC9990931; doi:10.1371/journal.pone.0276238)
Supplement: S2 Table — (DOCX) [file pone.0276238.s002.docx]

**S2 Table. The number and proportion of respondents who were exposed to all true and false statements from six round of surveys**

| **Round** | **No.** | **Statement** | **T/F** | **Generalizability knowledge statement** | **Number of respondents who were exposed to statement** | **Number of respondents who were not exposed to statement** | **Total** |
| --- | --- | --- | --- | --- | --- | --- | --- |
|  | **1** | Vaccinated people will not become infected with COVID-19 in the future | F | Yes | 13,500 (26.4) | 37,724 (73.6) | 51,224 (100.0) |
|  | **2** | After the injection, you should wait to observe the side effects and severe symptoms mostly present within 30 minutes | T | Yes | 23,378 (45.6) | 27,846 (54.4) | 51,224 (100.0) |
| **round 1** | **3** | Vaccines used in Thailand have to be approved by the National Vaccine Institute | F | No | 20,794 (40.6) | 30,430 (59.4) | 51,224 (100.0) |
|  | **4** | The Ministry of Public Health negotiated with various vaccine companies without conflict of interest with specific companies | T | No | 18,326 (35.8) | 32,898 (64.2) | 51,224 (100.0) |
|  | **5** | Health professionals, elderly and patients with chronic diseases are a priority in the vaccination plan | T | No | 29,641 (57.9) | 21,583 (42.1) | 51,224 (100.0) |
|  | **1** | Aztrazeneca was approved as benefits outweigh the possible risks | T | Yes | 38,538 (73.0) | 14,287 (27.0) | 52,825 (100.0) |
|  | **2** | People who want to get vaccinated, can pay instead of queuing | F | No | 16,987 (32.2) | 35,838 (67.8) | 52,825 (100.0) |
| **round 2** | **3** | Side effects such as headache, fever, nausea are common symptoms after vaccination | T | Yes | 40,860 (77.3) | 11,965 (22.7) | 52,825 (100.0) |
|  | **4** | If you are fully vaccinated, it is not necessary to observe social distancing, wearing mask and washing hands | F | Yes | 15,432 (29.2) | 37,393 (70.8) | 52,825 (100.0) |
|  | **5** | Sinovac has been approved by Thai Food and drug administration before distributing to people | T | No | 40,045 (75.8) | 12,780 (24.2) | 52,825 (100.0) |
|  | **1** | “Mhor Prom” application can use for vaccine registration, monitoring side effects and getting vaccine certification | T | No | 24,602 (91.8) | 2,193 (8.2) | 26,795 (100.0) |
|  | **2** | Pregnant (gestational age more than 12 weeks) or breastfeeding woman can get the vaccine | T | Yes | 17,976 (67.1) | 8,819 (32.9) | 26,795 (100.0) |
| **round 3** | **3** | Sinovac vaccine causes permanent effects of stroke | F | Yes | 14,621 (54.6) | 12,174 (45.4) | 26,795 (100.0) |
|  | **4** | Vaccines used in Thailand (Sinovac & Aztrazeneca) have low efficacy in preventing severe illness and death | F | Yes | 17,509 (65.3) | 9,286 (34.7) | 26,795 (100.0) |
|  | **5** | If there are any adverse events from the vaccine, you will be reimbursed by the National Health Security Office | T | No | 21,461 (80.1) | 5,334 (19.9) | 26,795 (100.0) |
|  | **1** | The National Health Security Office will reimburse all people who have side effects from vaccines before investigation | T | No | 18,801 (72.2) | 7,236 (27.8) | 26,037 (100.0) |
|  | **2** | Women who take contraceptive pills do not need to stop before the vaccination | T | Yes | 16,324 (62.7) | 9,713 (37.3) | 26,037 (100.0) |
| **round 4** | **3** | After being fully vaccinated, people will have a lifelong immunity level | F | Yes | 9,811 (37.7) | 16,226 (62.3) | 26,037 (100.0) |
|  | **4** | The number of distributed vaccines in each province depends on the number of registered people | F | No | 18,048 (69.3) | 7,989 (30.7) | 26,037 (100.0) |
|  | **5** | People who are allergic to foods or have allergic rhinitis can get the vaccine | T | Yes | 17,985 (69.1) | 8,052 (30.9) | 26,037 (100.0) |
|  | **1** | If you have already had a COVID-19 infection and are already cured, you should also get the vaccine | T | Yes | 18,856 (85.0) | 3,318 (15.0) | 22,174 (100.0) |
|  | **2** | People who drink coffee and tea regularly do not need to stop before vaccination | T | Yes | 16,281 (73.4) | 5,893 (26.6) | 22,174 (100.0) |
| **round 5** | **3** | You should not receive an anesthetic injection after the vaccination within one month so you cannot have a dental procedure | F | Yes | 8,931 (40.3) | 13,243 (59.7) | 22,174 (100.0) |
|  | **4** | The mRNA vaccine will introduce DNA mutations | F | Yes | 8,130 (36.7) | 14,044 (63.3) | 22,174 (100.0) |
|  | **5** | If there are no side effects after vaccination, it means no or low level of immunity | F | Yes | 11,520 (52.0) | 10,654 (48.0) | 22,174 (100.0) |
|  | **1** | If you have low immunization level after vaccination, it means no immunity | F | Yes | 8,724 (59.4) | 5,965 (40.6) | 14,689 (100.0) |
|  | **2** | You should not take paracetamol after vaccination because it affects vaccines | F | Yes | 4,045 (27.5) | 10,644 (72.5) | 14,689 (100.0) |
| **round 6** | **3** | There is a recommendation to switch vaccines with the 1^st^ dose of Sinovac and 2^nd^ dose of Astrazeneca | T | No | 12,754 (86.8) | 1,935 (13.2) | 14,689 (100.0) |
|  | **4** | “Mhor Prom” application is open for children age 12-18 years old to register for vaccination | F | No | 8,239 (56.1) | 6,450 (43.9) | 14,689 (100.0) |
|  | **5** | Thai people who get alternative vaccines organized by private hospitals, have to claim for side effects from those hospitals | T | No | 8,386 (57.1) | 6,303 (42.9) | 14,689 (100.0) |

Note: False statements (F); True statements (T)
