# Supplementary material for: The impact of vaccine information and other factors on COVID-19 vaccine acceptance in the Thai population
Source: PLoS One. 2023 Mar 7;18(3):e0276238. doi: 10.1371/journal.pone.0276238 (PMC9990931; doi:10.1371/journal.pone.0276238)
Supplement: S3 Table — (DOCX) [file pone.0276238.s003.docx]

**S3 Table. Questionnaire**

**Module 1: Demography**

| 1. Age _____ years  2. Sex   - Male - Female - Prefer not to say   3. Religious   - Buddhist - Christian - Muslim - Non-religious - Others, please specify …………………………………   4. Highest education level   - Below elementary school - Elementary school - High school / vocational certificate / equivalence - High vocational certificate / equivalence - Bachelor’s degree / equivalence - Master’s degree or higher   5. Occupation   - Bureaucrat/state enterprise officer/civil servant/government officer (except medical or healthcare worker) - Private company’s employer (except medical or healthcare worker) - Medical or healthcare worker - Business owner/merchant - Freelance - Public transportation driver (bus, train, taxi, motor-tricycle or motorbike) - Daily labourer - Messenger/delivery services - Agriculture - Student - Unemployed/retired   6. Are you the Village Health Volunteer?   - Yes - No   7. Province you are currently living   - Choice of 77 provinces for selection   8. Do you have any chronic diseases? (Can choose more than one answer)   - Diabetes (both type 1 and type 2) - Cardiovascular disease (including high blood pressure and hyperlipidemia) - Neurovascular disease - Chronic kidney disease (end stage) - Chronic respiratory disease e.g. chronic obstructive pulmonary disease (COPD) and uncontrolled asthma - Cancer - Obesity (weight > 100 kilograms or BMI > 35 kg/m2) - Others, please specify ………………………………… - None |
| --- |

**Module 2: Vaccine acceptance**

| 1. Do you want to get the Covid-19 vaccine? (Please choose only one answer)  - Yes - No - Not sure |
| --- |

**Module 3: Exposure and ability to differentiate** **true/false information**

| 1. Have you ever seen these statements?  \| **Statement** \| **Have seen** \| **Have not seen** \| \| --- \| --- \| --- \| \| [statement1] \|  \|  \| \| [statement2] \|  \|  \| \| [statement3] \|  \|  \| \| [statement4] \|  \|  \| \| [statement5] \|  \|  \|  1. Do you think these statements are true or false?  \| **Statement** \| **True** \| **False** \| **Not sure** \| \| --- \| --- \| --- \| --- \| \| [statement1] \|  \|  \|  \| \| [statement2] \|  \|  \|  \| \| [statement3] \|  \|  \|  \| \| [statement4] \|  \|  \|  \| \| [statement5] \|  \|  \|  \| |
| --- | --- | --- | --- | --- | --- | --- | --- | --- | --- | --- | --- | --- | --- | --- | --- | --- | --- | --- | --- | --- | --- | --- | --- | --- | --- | --- | --- | --- | --- | --- | --- | --- | --- | --- | --- | --- | --- | --- | --- | --- | --- | --- |

**Module 4: Trustworthy sources of information**

| 1. To what extent do you trust these sources of information?  \| **Sources of information** \| **1**  **(least trust)** \| **2** \| **3** \| **4** \| **5**  **(most trust)** \| \| --- \| --- \| --- \| --- \| --- \| --- \| \| Health professional \|  \|  \|  \|  \|  \| \| Academia \|  \|  \|  \|  \|  \| \| Village Health Volunteer \|  \|  \|  \|  \|  \| \| Close person \|  \|  \|  \|  \|  \| \| Government \|  \|  \|  \|  \|  \| \| Media \|  \|  \|  \|  \|  \| \| Public figure \|  \|  \|  \|  \|  \| |
| --- | --- | --- | --- | --- | --- | --- | --- | --- | --- | --- | --- | --- | --- | --- | --- | --- | --- | --- | --- | --- | --- | --- | --- | --- | --- | --- | --- | --- | --- | --- | --- | --- | --- | --- | --- | --- | --- | --- | --- | --- | --- | --- | --- | --- | --- | --- | --- | --- |

**Module** **5**: **Other factors related to vaccine acceptance**

| 1. How would you rate your chance of becoming infected with COVID-19? (1 means lowest risk and 5 means highest risk) 2. To what extent do you agree with these sentences?  \| **Statement** \| **1**  **(totally disagree)** \| **2**  **(disagree)** \| **3**  **(neutral)** \| **4**  **(agree)** \| **5**  **(totally agree)** \| \| --- \| --- \| --- \| --- \| --- \| --- \| \| COVID -19 vaccines are safe \|  \|  \|  \|  \|  \| \| COVID -19 vaccines are effective \|  \|  \|  \|  \|  \| \| COVID -19 vaccines are important to me \|  \|  \|  \|  \|  \| \| I trust the government \|  \|  \|  \|  \|  \| \| I trust health providers and health system \|  \|  \|  \|  \|  \| \| I trust vaccine producers \|  \|  \|  \|  \|  \| |
| --- | --- | --- | --- | --- | --- | --- | --- | --- | --- | --- | --- | --- | --- | --- | --- | --- | --- | --- | --- | --- | --- | --- | --- | --- | --- | --- | --- | --- | --- | --- | --- | --- | --- | --- | --- | --- | --- | --- | --- | --- | --- | --- |
